# A Diffused Mini-Sniffing Sensor for Monitoring SO_2_ Emissions Compliance of Navigating Ships

**DOI:** 10.3390/s22145198

**Published:** 2022-07-12

**Authors:** Mengtao Deng, Shitao Peng, Xin Xie, Zhi Jiang, Jianbo Hu, Zhaoyu Qi

**Affiliations:** 1 Key Laboratory of Environmental Protection Technology on Water Transport, Ministry of Transport, National Engineering Research Center of Port Hydraulic Construction Technology, Tianjin Research Institute for Water Transport Engineering, M.O.T., Tianjin 300456, China; dengmt@tiwte.ac.cn (M.D.); hujb@tiwte.ac.cn (J.H.); qizhy@tiwte.ac.cn (Z.Q.); 2 Shanghai Maritime Safety Administration of the People’s Republic of China, Shanghai 200086, China; xiexin@shmsa.gov.cn; 3 Baoshan Maritime Safety Administration of the People’s Republic of China, Shanghai 201999, China; jiangzhi@shmsa.gov.cn

**Keywords:** UAV, emission control areas, diffused mini-sniffing sensor, ship exhaust, fuel sulfur content

## Abstract

The ship exhaust sniffing unmanned aerial vehicle (UAV) system can be applied to monitor vessel emissions in emission control areas (ECAs) to improve the efficiency of maritime law enforcement and reduce ship pollution. To solve the problems of large size, heavy weight and high cost of ship exhaust sniffing sensors, in this paper, a unique diffused mini-sniffing sensor was designed, which provides a low-cost, lightweight, and highly adaptable solution for ship exhaust sniffing UAV. To verify the measurement accuracy of the system, a large number of on-site tests were performed based in the mouth of the Yangtze River, and some cases of violation of the fuel sulfur content (FSC) were verified and punished. Maritime law enforcement officers boarded the ship to take oil samples from eight suspected ships and sent them to the laboratory for testing. The results showed that the FSCs of the eight ships in chemical inspection were all greater than the regulatory limit 0.5% (m/m) of the International Maritime Organization (IMO). The system enables authorities to monitor emissions using rotary UAVs equipped with diffused mini-sniffing sensors to measure the FSC of navigating ships, which couple hardware and operational software with a dedicated lab service to produce highly reliable measurement results. The system offers an effective tool for screening vessel compliance.

## 1. Introduction

The shipping industry is the artery of global trade and plays an important role in promoting the world economy and trade development and in stabilizing the global supply chain. According to the report of the United Nations Conference on Trade and Development, more than 70% of the current international trade volume and more than 80% of the international trade volume are realized through shipping [1,2]. In recent years, with the rapid development of the shipping industry, the problem of air pollution caused by ship emissions has become increasingly serious [3,4,5,6,7,8,9,10]. A large number of research results reveal that CO_2_, SO_2_ and NO_2_ levels emitted by ships account for 3%, 6% and 15% of all human emissions [3,11], respectively. SO_2_ emitted by fuel combustion with sulfur content exceeding the standard 0.5% (m/m) causes more than 60,000 premature deaths worldwide every year [12]. The use of this fuel is harmful to the coastal environment and port communities, and SO_2_ can form acid rain, acidify the water quality, cause changes in the water ecosystem, and endanger the growth of animals and plants. In 1997, the International Maritime Organization (IMO) formulated the MARPOL73/78 Annex VI, “Rules for the Prevention of Air Pollution from Ships”, which requires that the sulfur content of global marine fuel oil shall not exceed 3.5% (m/m) from 2012 and 0.5% (m/m) after 2020 [13,14].

For reducing the impact of ship exhaust on air quality in coastal cities, developed countries in Europe and the United States have set up air pollutant ship emission control areas (ECAs) with stricter standards. Since 2015, ships entering ECAs are required to use fuel with sulfur content of no more than 0.1% (m/m). Compared with developed countries in Europe and the United States, China’s coastal areas have more developed water transportation and a higher population density. The damage of ship exhaust to the health of coastal residents is significantly more serious, which has attracted the attention of the Chinese government. To reduce air pollution caused by ship exhaust emissions, in December 2015, The Ministry of Transport (MOT) of China issued an Action Plan, which was used to control marine emissions in the Bohai-rim Waters, Yangtze River Delta and Pearl River Delta region [15]. For the purpose of reducing the emissions of SO_2_, NO_x_ and PM from ships, on 10 December 2018, the MOT issued the “Implementation Scheme of the Domestic Emission Control Areas (DECAs)” for Atmospheric Pollution from Vessels [16,17,18]. Therefore, ship emission regulation has become an important work in daily law enforcement of the maritime system.

In view of the emission supervision of ships underway, the traditional direct boarding inspection method has the problems of difficult boarding, low supervision efficiency, and low sampling inspection ratio. At present, most of the high-efficiency monitoring methods of ship exhaust without boarding use the sniffing method [19], which monitors the concentration relationship of SO_2_ and CO_2_ in the exhaust plume of the ship, so as to estimate the FSC. According to the location of installation, the monitoring equipment includes: a shore-based or bridge-based fixed ship exhaust remote monitoring instrument and a boat-borne ship exhaust telemeter and airborne ship exhaust telemeter [19,20,21,22,23,24,25,26]. Among them, the airborne ship exhaust telemeter cannot only overcome the influence of fixed wind direction conditions and monitoring distance, it can also solve the problems of low efficiency and high cost of boat-borne monitoring.

Additionally, many researchers have successfully used small aircraft equipped with sensors to monitor navigating ships. The European Union Joint Research Center JRC launched a sister project of the SIRENAS-R project, the SIRENAS-G project, in conjunction with the Italian Coast Guard, which carried out experiments in the Gulf of Genoa using unmanned aerial vehicles (UAV) equipped with small SO_2_- and CO_2_-monitoring instruments and equipment. In the experiment, the tail gas of 10 ships was monitored [27]. From the results, the UAV sniffing method had a better signal-to-noise ratio because of the close contact with the exhaust. Beecken et al. [19] used small aircraft to carry sensors to monitor navigating ships in ECAs. However, the monitoring of navigating ships is relatively rare. Mellqvist et al. [28] used aircraft-mounted sensors to monitor ship exhaust. During the 27-h flight, 114 ships were monitored, and the cost of monitoring each ship was about EUR 470, regardless of other costs. Therefore, the use of aircraft-borne sensors to monitor the exhaust emissions of navigating ships has high cost and cannot widely monitor the emissions of ships. In addition, it may not always be feasible to measure near the pollution source, and it may be too dangerous or risky for someone to fly close to the ground [29]. Villa et al. [12] used an unmanned aerial vehicle (UAV) to characterize the gaseous (CO_2_) and particle (10–500 nm) emissions of a ship at sea, the UAV system successfully assessed ship emissions to derive EF_PN_ under real world conditions. Ning et al. [30,31] developed a practical alternative method to collect emissions and to determine FSC, using UAV aircraft equipped with compact air pollution-monitoring sensors to monitor ship exhaust emissions. Zhou et al. [17,18] designed and developed a set of UAV systems to monitor the emissions of navigating ships and achieved good experimental results. Compared with aircraft, the method of using UAV to carry sniffing sensors to monitor tail gas has the advantages of small size, low cost and simple operation. However, at present, the UAV airborne ship exhaust monitoring instrument used in the market is large, heavy and expensive, and a suitable UAV is fixed and has a high cost, which cannot meet the needs of wide application based on a maritime system. Simultaneously, maritime law enforcement personnel have the current problem of “dare not fly and can’t fall”. The application range for UAVs for a ship exhaust sniffing system is greatly limited.

Based on the above problems, a unique diffused mini-sniffing sensor was designed, which provides a low-cost, lightweight, and highly adaptable solution for a ship exhaust sniffing UAV. The UAV is equipped with a diffused mini-sniffing sensor to fly into the ship’s tail gas plume for monitoring. By analyzing the ratio of SO_2_ and CO_2_, it can quickly analyze whether the FSC exceeds the standard without boarding. A large number of on-site tests were carried out in the mouth of the Yangtze River, and some cases of violation of the FSC were verified and punished, which verified the reliability and accuracy of the diffused mini-sniffing sensor.

## 2. Materials and Methods

**Figure 1:**
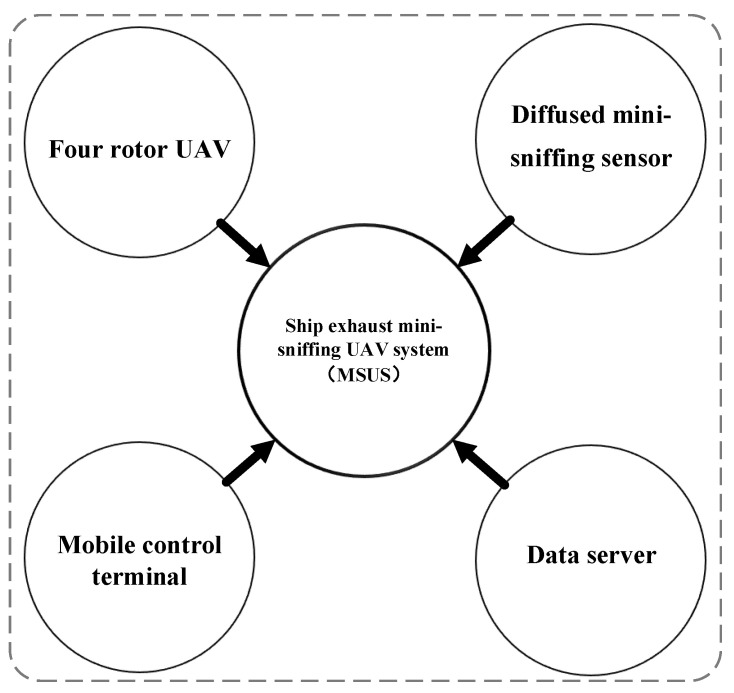
The structure of ship exhaust mini-sniffing UAV system.

The research undertaken in this paper is from the “Monitoring ship exhaust emissions of navigating ships in Key waters of Shanghai” project. In this project, a set of ship exhaust mini-sniffing UAV systems (MSUS) is designed, which is used for monitoring the FSC of navigating ships in ECAs. MSUS consists of four modules: (1) four rotor UAV; (2) diffused mini-sniffing sensor; (3) mobile control terminal; (4) data server. The system structure is shown in Figure 1. When the system is in normal operation, the remote-control UAV flies to the ship’s tail gas plume with a diffused mini-sniffing sensor, collects the concentration data of SO_2_ and CO_2_ in real time, records the time information, calculates the FSC, and completes the data integration and packaging process.

**Figure 2:**
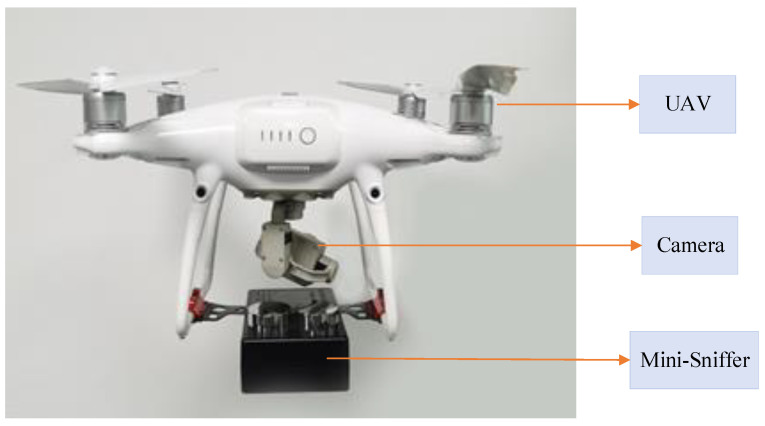
Image of ship exhaust mini-sniffing UAV system.

The DJI Phantom 4 Pro V2.0 UAV (SZ DJI Technology Co., Ltd., Shenzhen, China) is used as the flight carrier of the sniffing sensor, and a diffused mini-sniffing sensor is designed and manufactured, which is installed under the bottom bracket of the UAV, as shown in Figure 2. The camera and diffused mini-sniffing sensor are installed under the UAV. The camera is used to assist in finding the ship’s funnel mouth during flight. The diffused mini-sniffing sensor is used to carry sensors for SO_2_ and CO_2_, and the processor, battery and communication modules. MSUS has been tested in the key waters of Shanghai (ECA), which has verified the stability, reliability and safety of the system.

### 2.1. Diffused Mini-Sniffing Sensor

**Figure 3:**
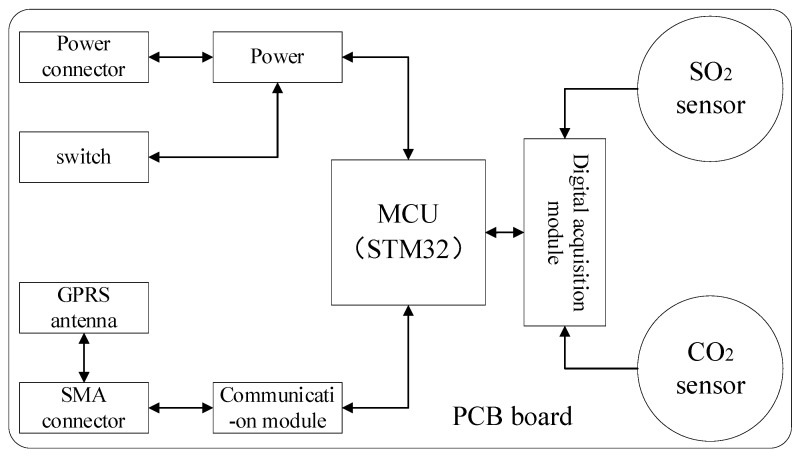
The structure diagram of the diffused mini-sniffing sensor.

As an important part of gas detection, the diffused mini-sniffing sensor mainly includes an SO_2_ sensor, CO_2_ sensor, power supply module, processor and communication module. The size of the diffused mini-sniffing sensor is 140 × 80 × 40 mm, weighing about 300 g. The structure diagram of the diffused mini-sniffing sensor is shown in Figure 3. During the monitoring process, the UAV flies into the plume of the tail gas with the sensor to make the tail gas react with the sensor, and the wireless communication module sends data to the receiving end on the ground in real time.

#### 2.1.1. CO_2_ Sensor

The boat-mounted ship exhaust telemeter monitors the exhaust gas of navigating ships. It is necessary to maintain a safe distance of more than 100 m between the sea patrol boat and the measured ship. The concentration of exhaust gas that can be monitored is low; thus, a high-precision CO_2_ sensor with a large volume and weight must be used. The ship exhaust sniffing UAV can carry the diffused mini-sniffing sensor to fly directly into the smoke plume for monitoring and analysis, and it can monitor the high concentration of tail gas within tens of meters. Therefore, the UAV airborne sniffer can sacrifice the accuracy of the sensor, so as to reduce the volume and weight of the sensor.

According to the test experience, the concentration range of CO_2_ after tens of meters of diffusion dilution of ship tail gas is about 100–1500 ppm (removing the concentration of CO_2_ in the air background). Therefore, this paper adopts a non-dispersive infrared CO_2_ sensor with small size, light weight and suitable precision. This kind of sensor has the advantages of excellent long-term stability and low maintenance cost. The CO_2_ sensor was purchased from Shenzhen Singoan Electronic Technology Co., Ltd., Shenzhen, China; sensor model: SGA-700B-CO_2_; technical principles: non-dispersive infrared. The selected CO_2_ sensor has a detection range of 0–2000 ppm, a detection accuracy of 5 ppm, a response time T_90_ less than 30 s, a sensor diameter of 34.5 mm, a height of 30 mm, and a weight of about 30 g.

#### 2.1.2. SO_2_ Sensor

Comprehensively consider the principle of the sniffing method: that is, the molar ratio of C and H of marine fuel is about 1:2, and the C content is calculated at about 87%. From the principle of material conservation, the S content in the fuel oil can be deduced by monitoring the ratio of SO_2_ and CO_2_ in the ship tail gas after diffusion dilution. The *FSC* can be calculated by Equation (1):

(1) $$FSC\left(\%\right)=\frac{S\left(\mathrm{kg}\right)}{\mathrm{Fuel}\left(\mathrm{kg}\right)}=\frac{{\mathrm{SO}}_{2}\left(\mathrm{ppb}\right)\times A\left(S\right)}{{\mathrm{CO}}_{2}\left(\mathrm{ppm}\right)\times A\left(C\right)}\times 87\%=\frac{{\mathrm{SO}}_{2}\left(\mathrm{ppb}\right)}{{\mathrm{CO}}_{2}\left(\mathrm{ppm}\right)}\times 0.232\%$$

where *FSC* is the sulfur content of the fuel, A(S) is the relative atomic mass of sulfur, and A(C) is the relative atomic mass of carbon.

According to the sniffing method, when the ratio of SO_2_ and CO_2_ in the ship’s tail gas reaches more than 3 times, which means the *FSC* exceeds 0.5% (m/m). In this paper, a SO_2_ sensor based on the electrochemical method is adopted. Because of its low power consumption, small size, light weight and high precision, an SO_2_ electrochemical sensor is widely used. The SO_2_ sensor was purchased from Shenzhen Singoan Electronic Technology Co., Ltd.; sensor model: SGA-700B-SO_2_; technical principles: electrochemical. The selected electrochemical SO_2_ sensor has a detection range of 0–1 ppm, a detection accuracy of 10 ppb, a response time T_90_ less than 30 s, a sensor diameter of 34.5 mm, a height of 30 mm, and a weight of about 30 g.

#### 2.1.3. Power-Supply Module

The power of the diffused mini-sniffing sensor is about 12W; thus, the power module can be powered by two No.5 batteries separately, which can not only reduce the secondary development cost of the UAV but can also be independent of the UAV and can be easy to disassemble. The capacity of a single battery is 2775 mAh, and the capacity of two batteries is 5550 mAh. According to 70% of the effective capacity, the effective capacity is 3885 mAh. The voltage of the two batteries is 3V, which can support the sensor to work continuously for about 1 h after calculations. Generally, the effective flight time of the rotor UAV does not exceed 30 min, and the power supply module can support the ship exhaust detection of the UAV’s normal endurance flight time.

### 2.2. UAV

Combined with the wind speed of the inland river emission control area, the weight of the sensor, and the endurance of the UAV, the DJI Phantom 4 Pro V2.0 UAV was selected as the flight carrier of the diffused mini-sniffing sensor. The system adopts phantom 4 Pro V2.0 UAV, which is the most popular consumer UAV at present. It has the advantages of small size, low cost and simple operation. The diffused mini-sniffing sensor can be fixed on the two brackets of the UAV, and the pan-tilt camera can transmit a real-time picture of the ship’s exhaust to the remote control for the technician to view.

Table 1 shows the key parameters of the UAV. The weight of the diffused mini-sniffing sensor is about 300 g. After carrying the sensor, the UAV can fly for about 25 min. Assuming that the ship flow is dense, start the UAV at the point close to the channel to monitor the exhaust emissions of the navigating ships. Using a UAV battery can complete at least 3–4 ship tail gas monitoring tasks. During the experimental test at the Wusongkou international cruise port in Shanghai, the experimental group prepared 6 UAV batteries for cyclic charging according to 8 h per day, which can meet 8 h continuous monitoring. When the weather and wind speed are appropriate (the appropriate wind speed is less than 10 m/s), the number of ships monitored every day can reach 60.

### 2.3. Data Server

The data server of the ship exhaust mini-sniffing UAV system is realized by a laptop and a data-receiving module. The software functions mainly include acquisition control, data display, FSC estimation and historical data query. Technicians can control the UAV to fly into the exhaust plume of navigating ships as well as remotely monitor the SO_2_ and CO_2_ concentration curves in real time through the data server to screen suspected ships that use high-sulfur oil in violation of regulations.

## 3. Experimental Methods

### 3.1. Monitoring Conditions

**Figure 4:**
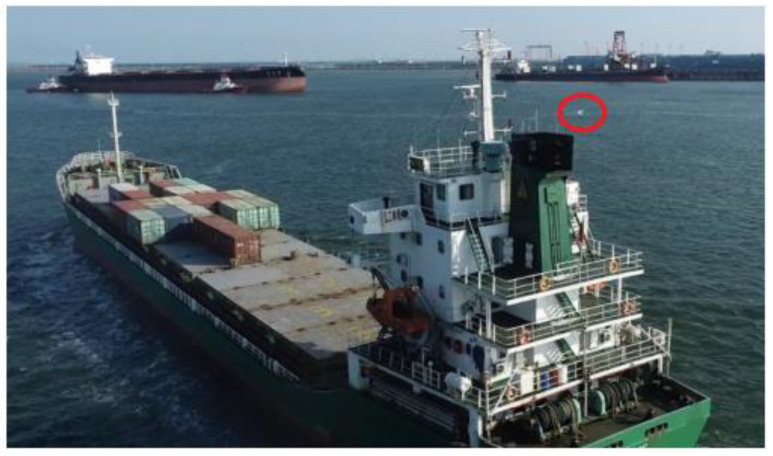
Image of UAV on-site tracking ship. The red ellipse indicates the actual monitoring position of the UAV.

When the wind speed is less than 10 m/s and the wind direction is different, the position of the UAV relative to the chimney is various. It should always be kept at a close distance to the downwind direction. Although the distance between the UAV and the ship’s chimney is different, and the concentrations of the exhaust components are different, the CO_2_ and SO_2_ are diffused and diluted with the wind, but the ratio remains unchanged. As long as the exhaust gas can be monitored, the FSC can be estimated. A safe distance between the UAV and the ship’s chimney should be 10–15 m, the height should be equal to or slightly higher than 1–2 m of the chimney height, and it should be followed for about 1–2 min. Figure 4 shows the image of the on-site experimental test of the ship exhaust sniffing UAV. The experimenters control the UAV to fly near the chimney of the tested ship and adjust the optimal measurement position of the UAV according to the wind speed and direction, so as to monitor the concentrations of SO_2_ and CO_2_.

### 3.2. Monitoring Method

The Yangtze River is the longest river in China, and the Yangtze River is a golden waterway with the largest freight volume in the world. The density of ships is high, especially at the mouth of the Yangtze River, where ships are constantly flowing. Therefore, the experimental test site was selected in the key waters of the Shanghai Emission Control Area, Yangtze River. The test site was preferred to take off on shore, which has the advantages of simple operation and low cost. The monitoring process of ship exhaust mini-sniffing UAV includes a series of steps.

1. Observe the relative distance between the ship and the monitoring point, select an appropriate take-off time, and control the ship exhaust mini-sniffing UAV to fly to the monitored ship;
2. Adjust the position of the UAV according to the wind speed, wind direction and the height of the ship’s chimney to ensure that the UAV is always in the downwind direction of the plume;
3. Control the position of the UAV in the downwind direction of the plume, keep a safe distance of 10–15 m from the ship’s chimney, and track and monitor for 1–2 min;
4. Check the concentrations of SO_2_ and CO_2_ in the curve of the data-monitoring software in real time, and determine whether the FSC exceeds the standard according to the FSC estimated by the software;
5. After the exhaust gas monitoring is completed, observe whether the power of the drone is sufficient. If the power is sufficient, continue to track and monitor the next ship; if the power is low, control the UAV to return.

### 3.3. Calculation of FSC

The carbon content in marine fuel oil or diesel oil is about 87%. Under ideal conditions of the engine, all carbon and sulfur are burned and converted into CO_2_ and SO_2_, respectively. Since the CO_2_ and SO_2_ sensors in this paper have the same response time (T_90_ less than 30 s) and the same sampling frequency (the data sampling frequency is set to 1 s), the peak concentration of SO_2_ and CO_2_ is used to calculate FSC [20,24,32,33]. The sulfur content in the fuel can be calculated by Equation (1). The real-time calculation and processing of the CO_2_ and SO_2_ concentration data detected by the sniffing sensor through the micro-controller can estimate the FSC in real time and send the FSC data to the server through the communication module.

## 4. Experimental Results and Analysis

### 4.1. Comparative Analysis of Ship Exhaust Sniffing UAV

**Figure 5:**
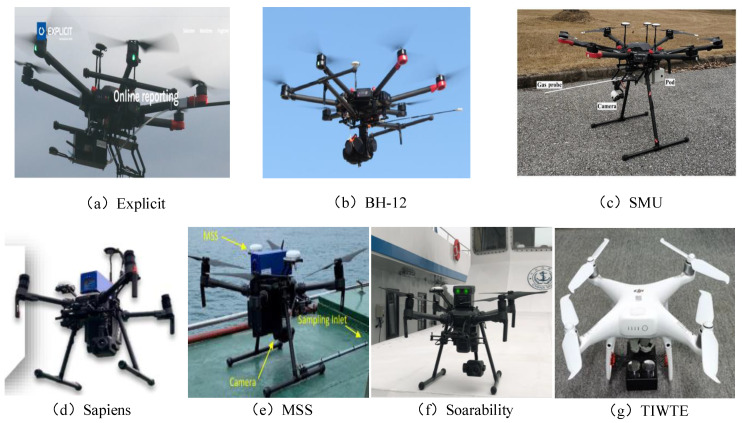
Comparison of different ship exhaust sniffing UAVs.

Figure 5 shows the of ship exhaust sniffing UAVs of different units, including Explicit [34], BH-12 [34], SMU [18], Spaiens [34], MSS [35], Soarability [29] and TIWTE, and Table 2 shows the parameter comparison between several ship exhaust sniffing UAVs and the ship exhaust mini-sniffing UAV proposed in this paper. DJI phantom 4 Pro was used as the flight carrier of the miniature sniffing sensor in the research.

As can be seen from Figure 5 and Table 2, compared with other ship exhaust sniffing UAVs, the ship exhaust mini-sniffing UAV (Figure 5g) proposed in this paper has multiple advantages in ship exhaust monitoring. First, the diffused mini-sniffing sensor adopts a diffuse design structure; compared with the pumping structure, the air circuit module (including the intake pipe, filter, air pump, etc.) is abandoned. The design structure in this paper can not only reduce the weight of the sniffing sensor but can also reduce the volume of the sensor. Second, the diffused mini-sniffing sensor weighs about 300 g, which has the advantages of easy installation and portability. Finally, this paper adopts DJI Phantom 4 Pro V2.0 UAV, which has the advantages of small size, light weight, low cost, easy portability and fast charging. The system has undergone a large number of experimental tests to verify the stability, reliability and safety of the ship exhaust mini-sniffing UAV from many aspects.

### 4.2. Accuracy Analysis

**Figure 6:**
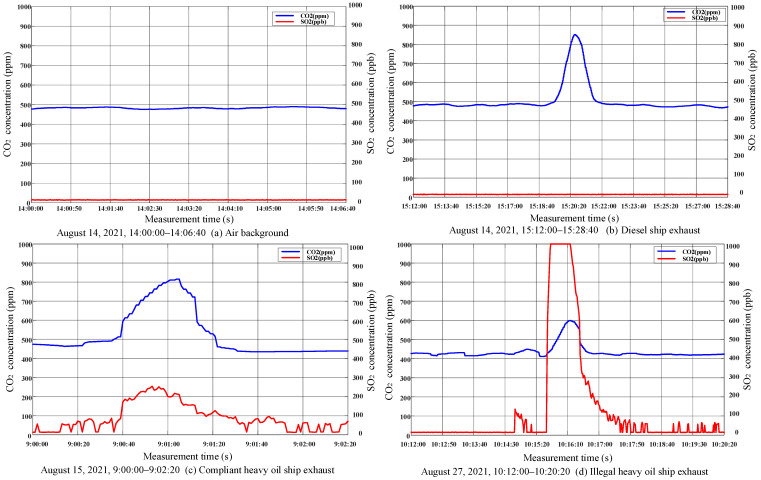
Monitoring curve of sulfur content in different fuel.

To verify the effectiveness of the diffused mini-sniffing sensor, the experimental group conducted a comparative test on the ship exhaust of different sulfur content fuels. Figure 6 shows the measurement results of the ship exhaust of different sulfur content fuels, and the changes in SO_2_ and CO_2_ concentrations are shown in the monitoring curve. Figure 6a–d successively shows the concentration curves of air background, diesel ship exhaust, compliant heavy oil ship exhaust and ship exhaust with excessive sulfur content. The sampling interval in the four experiments was 1 s, but the effective collection time (that is, when the concentrations of SO_2_ and CO_2_ were actually measured) was different for each plume: (a) air background, 14:00:00–14:06:40, 14 August 2021; (b) diesel ship exhaust, 15:12:00–15:28:40, 14 August 2021; (c) compliant heavy oil ship exhaust, 9:00:00–9:02:20, 15 August 2021; (d) illegal heavy oil ship exhaust, 10:12:00–10:20:20, 27 August 2021.The X-axis and Y-axis of the curve represent time (s) and gas concentration, respectively, the red curve represents SO_2_ concentration (ppb), and the blue curve represents CO_2_ concentration (ppm). From Figure 6a–d, when the diffused mini-sniffing sensor monitors the air, the SO_2_ and CO_2_ concentrations basically do not change. When the diffused mini-sniffing sensor monitors the ship tail gas without sulfur, there will be obvious peaks of CO_2_, and the concentration of SO_2_ will remain basically unchanged. When the diffused mini-sniffing sensor monitors the ship tail gas of compliant low sulfur oil, there will be obvious peaks of CO_2_ and SO_2_. When the sniffing sensor monitors the ship tail gas of illegal high sulfur oil, it can be observed that the concentration of SO_2_ and CO_2_ will increase, and the increase in SO_2_ concentration is much greater than that in CO_2_.

**Figure 7:**
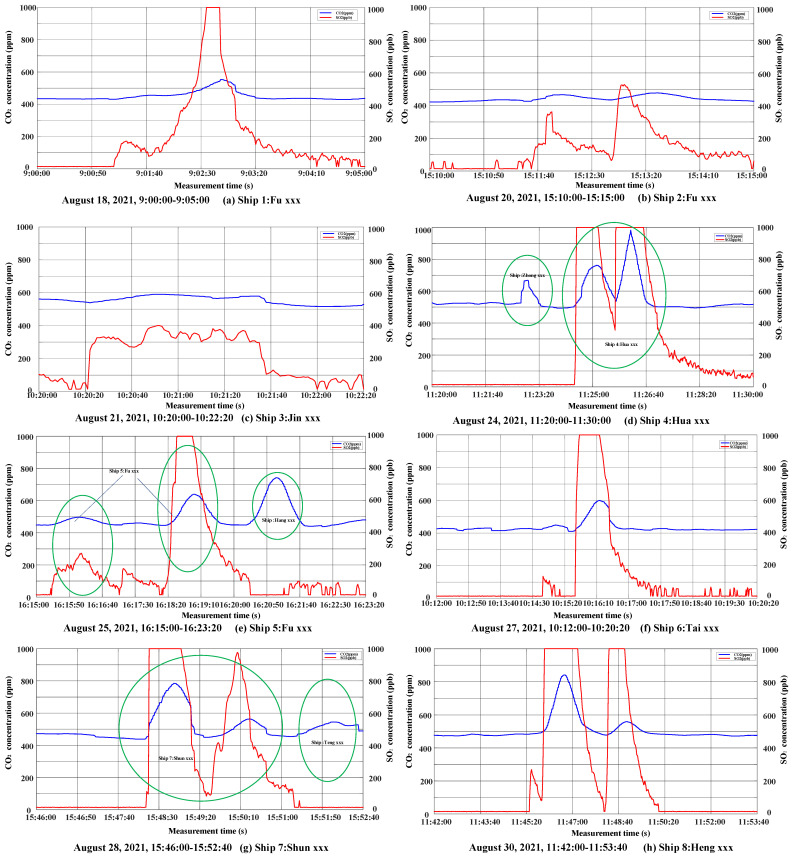
Monitoring curve of excessive sulfur content in ship fuel.

The test results show that when the increase in SO_2_ concentration in tail gas exceeds three times that of CO_2_ concentration, it can be considered that the FSC of the ship exceeds the standard. Figure 7 shows the actual monitoring curves of tail gas of the eight groups of high sulfur oil ships. The X-axis and Y-axis of the curve represent time and gas concentration, respectively, the red curve represents SO_2_ concentration (ppb), and the blue curve represents CO_2_ concentration (ppm). From Figure 7a–h, it can be seen that the increase in SO_2_ concentration in tail gas of high sulfur oil ships is more than three times that in CO_2_ concentration. Therefore, the sulfur content in ship fuel can be estimated according to the concentration changes of SO_2_ and CO_2_ in the monitoring curve. The peaks of (a), (c) and (f) in Figure 7 represent the exhaust gas monitoring results of ship 1, ship 3 and ship 6, respectively. The exhaust gas monitoring results of ships 1, 3 and 6 all exceeded 0.5% (m/m). The two peaks of (b) and (h) in Figure 7 represent the exhaust gas monitoring results of the same ship twice. The three peaks of (d), (e) and (g) in Figure 7 represent the exhaust gas monitoring results of two different ships. In Figure 7d, the first peak represents the exhaust gas monitoring result of the diesel ship: Zhong xxx. The second and third peaks indicate the monitoring result of the exhaust gas of ship 4, whose fuel sulfur content exceeds 0.5%(m/m). In Figure 7e, the first two peaks represent the monitoring result of the exhaust gas of ship 5, whose fuel sulfur content exceeds 0.5% (m/m), and the third peak represents the exhaust gas monitoring result of the diesel ship: Hang xxx. In Figure 7g, the first two peaks represent the monitoring result of the exhaust gas of ship 7, whose fuel sulfur content exceeds 0.5% (m/m), and the third peak represents the exhaust gas monitoring result of the diesel ship: Teng xxx.

To fully verify the accuracy of the sensor, we provided the comparison results between the measured value and the true value of FSC of eight ships. The true value of FSC is obtained through laboratory testing. The data are shown in Table 3.

**Figure 8:**
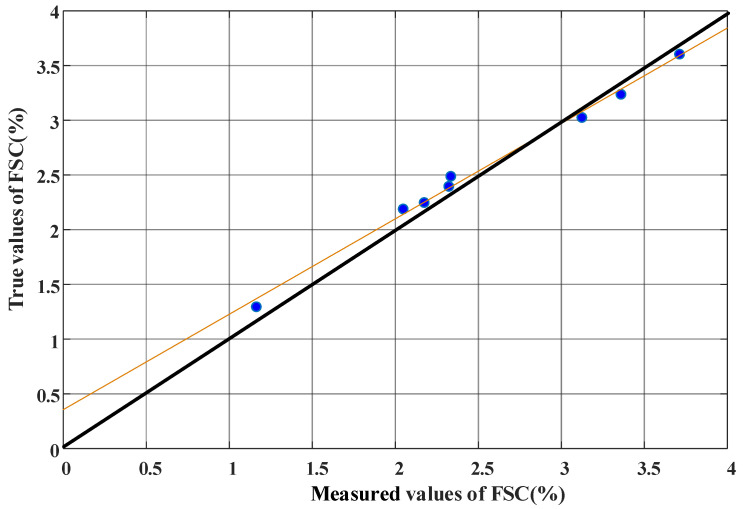
Comparison between the true value of FSC (X-axis) and the measured value of FSC (Y-axis) of 8 ships. The orange, black lines and blue dots represent the actual fitted curve, theoretical fitted curve and measured value of FSC, respectively.

From Table 3 and Figure 8 in the experimental test, the FSC of over-standard ships is mainly distributed between 1% (m/m) and 4% (m/m). In Table 3, error range in % means the difference between measured and true value divided by true value. The measured values of ships 1–8 in Table 3 correspond one-to-one with the exhaust gas monitoring results of (a–h) in Figure 7. From the analysis of the results in Table 3 and Figure 8, it can be seen that, to a certain extent, the measured values from UAV can be used to determine the sulfur content of ship fuels. The deviation may be caused by the accuracy of the sensor, the uncertainty of tail gas emission (not all sulfur in the fuel is discharged in the form of SO_2_), monitoring distance, and other factors. Although there is a certain deviation between the estimated FSC of the ship exhaust mini-sniffing UAV and the true FSC of the oil sample, it is basically within the allowable deviation ±0.2% (m/m), and the suspected ship locked by the UAV is a ship with excessive FSC, which verifies the reliability of the ship exhaust sniffing UAV technology based on the sniffing method. Through the analysis of the measured values of eight ships and the true values of laboratory tests, it can be concluded that the ship exhaust telemetry UAV proposed is effective for monitoring the illegal use of high-sulfur oil ships. In most cases, the estimated FSC is less than the true value; thus, the appropriate threshold and confidence interval can be set, and the estimated value can be used as the basis for the preliminary judgment of whether the ship exceeds the standard, which can establish a new supervision mode of “telemetry first and then boarding verification” for the maritime system.

## 5. Conclusions

To solve the problems of large size, heavy weight and high cost of the ship exhaust sniffing sensors on the market, in this paper, a diffused mini-sniffing sensor was designed, which provides a low-cost, lightweight, and highly adaptable solution for a ship exhaust sniffing UAV. From the test results, the reliability of ship exhaust sniffing UAV technology based on the sniffing method is fully verified. A new supervision mode of “telemetry first and then boarding inspection” can be established, which can improve the sampling inspection rate and reduce the supervision cost.

The ship exhaust mini-sniffing UAV system still has some parts that need to be improved. For example, the system is not suitable for complex weather such as rain and fog, and due to the limitation of wind resistance level, the system is not mature enough for the tail gas monitoring of navigating ships in the sea area, which still needs further research. In addition, the wireless communication module of the system adopts a single-chip wireless transceiver chip, which is not suitable for long-distance transmission and is blocked by obstacles, and the data receiver cannot obtain real-time monitoring results.

## Figures and Tables

**Table 1:** The key parameters of phantom 4 Pro V2.0 UAV.

Parameter	Value
Weight	1375 g
Battery capacity	5870 mAh
Dimensions	289.5 mm × 289.5 mm × 196 mm
Maximum rising speed	P-GPS: 5 m/s
Maximum descending speed	P-GPS: 3 m/s
Maximum horizontal flight speed	P-GPS: 50 km/h
Maximum flight altitude	6000 m
Maximum flight distance	8000 m
Maximum tolerable wind speed	10 m/s
Maximum flight time	30 min

**Table 2:** Comparison of parameters of different ship exhaust sniffing UAV.

Manufacturer	UAV Model	Flight Time (min)	Sensor Size (mm)	Sensor Weight (g)
Explicit	/	/	145 × 75 × 63	500
Aeromon Oy	DJI M600	40	182 × 180 × 200	960
SMU	DJI M600 PRO	40	200 × 120 × 90	900
Spaiens	DJ M210	35	170 × 66 × 66	750
MSS	DJ M210 RTK	35	195 × 75 × 75	750
Soarability	DJ M210	35	157 ×103 × 87	400–500
TIWTE	DJ Phantom 4	30	140 × 80 × 40	300

**Table 3:** Comparison between the true value of FSC and the measured value of FSC of 8 ships.

Number	Ship	Measured Value (m/m)	True Value (m/m)	Deviation (m/m)	Error Range
1	Fu xxx	2.32%	2.40%	−0.08%	3.33%
2	Fu xxx	2.05%	2.19%	−0.14%	6.80%
3	Jin xxx	2.33%	2.49%	−0.16%	6.21%
4	Hua xxx	3.36%	3.24%	+0.12%	3.46%
5	Fu xxx	3.71%	3.60%	+0.11%	2.97%
6	Tai xxx	1.16%	1.30%	−0.14%	10.26%
7	Shun xxx	2.17%	2.25%	−0.08%	3.33%
8	Heng xxx	3.12%	3.03%	+0.09%	3.22%

## Data Availability

The data presented in this study are available on request from the corresponding author. The data are not publicly available due to privacy.

## References

[1] (2020). ICS: Impact Of COVID-19 and the Intensifying Crew Change Crisis–2020 Annual Review. https://www.ics-shipping.org/publication/annual-review-2020/.

[2] UNCTAD: World Seaborne Trade by Types of Cargo and by Group of Economies, Annual, United Nations Conference on Trade and Developmen. https://unctadstat.unctad.org/wds/TableViewer/tableView.aspx?ReportId=32363.

[3] Eyring V., Isaksen I.S.A., Berntsen T., Collins W.J., Corbett J.J., Endresen O., Grainger R.G., Moldanova J., Schlager H., Stevenson D.S. (2010). Transport impacts on atmosphere and climate: Shipping. Atmos. Environ..

[4] Gencarelli C.N., Hedgecock I.M., Sprovieri F., Schürmann G.J., Pirrone N. (2014). Importance of Ship Emissions to Local Summertime Ozone Production in the Mediterranean Marine Boundary Layer: A Modeling Study. Atmosphere.

[5] Lee D.S., Pitari G., Grewe V. (2010). Transport impacts on atmosphere and climate: Aviation. Atmos. Environ..

[6] Liu H., Fu M.L., Jin X.X., Shang Y., Shindell D., Faluvegi G., Shindell C., He K.B. (2016). Health and climate impacts of ocean-going vessels in East Asia. Nat. Clim. Change.

[7] Sofiev M., Winebrake J.J., Johansson L. (2018). Cleaner fuels for ships provide public health benefits with climate trade-offs. Nat. Commun..

[8] Viana M., Hammingh P., Colette A., Querol X., Degraeuwe B., de Vlieger I., van Aardenne J. (2014). Impact of maritime transport emissions on coastal air quality in Europe. Atmos. Environ..

[9] Wang X., Shen Y., Lin Y., Pan J., Zhang Y., Louie P.K.K., Li M., Fu Q. (2019). Atmospheric pollution from ships and its impact on local air quality at a port site in Shanghai. Atmos. Chem. Phys..

[10] Yang M., Bell T.G., Hopkins F.E., Smyth T.J. (2016). Attribution of atmospheric sulfur dioxide over the English Channel to dimethyl sulfide and changing ship emissions. Atmos. Chem. Phys..

[11] Eyring V., Köhler H.W., van Aardenne J., Lauer A. (2005). Emissions from international shipping: 1. The last 50 years. J. Geophys. Res..

[12] Villa T.F., Brown R.A., Jayaratne E.R., Gonzalez L.F., Morawska L., Ristovski Z.D. (2019). Characterization of the particle emission from a ship operating at sea using an unmanned aerial vehicle. Atmos. Meas. Tech..

[13] IMO: Revised MARPOL Annex IV. 2008. https://www.bahamasmaritime.com/wp-content/uploads/2021/12/MN059-MARPOL-Annex-IV-Sewage-Pollution-Prevention-v1.0.pdf.

[14] (2016). International Maritime Organization (IMO): Sulphur Oxides (SOx) and Particulate Matter (PM)–Regulation 14. https://www.imo.org/en/OurWork/Environment/Pages/Sulphur-oxides-(SOx)-%E2%80%93-Regulation-14.aspx.

[15] MOT Implementation of the Ship Emission Control Area in Pearl River Delta, the Yangtze River Delta and the Bohai Rim (Beijing–Tianjin–Hebei Area), Ministry of Transport, C., Beijing, China, 2015. http://www.gov.cn/xinwen/2015-12/04/content_5019932.htm.

[16] Feng J., Zhang Y., Li S., Mao J., Patton A.P., Zhou Y., Ma W., Liu C., Kan H., Huang C. (2019). The influence of spatiality on shipping emissions, air quality and potential human exposure in the Yangtze River Delta/Shanghai, China. Atmos. Chem. Phys..

[17] Zhou F., Pan S., Chen W., Ni X., An B. (2019). Monitoring of compliance with fuel sulfur content regulations through unmanned aerial vehicle (UAV) measurements of ship emissions. Atmos. Meas. Tech..

[18] Zhou F., Hou L., Zhong R., Chen W., Ni X., Pan S., Zhao M., An B. (2020). Monitoring the compliance of sailing ships with fuel sulfur content regulations using unmanned aerial vehicle (UAV) measurements of ship emissions in open water. Atmos. Meas. Tech..

[19] Beecken J., Mellqvist J., Salo K., Ekholm J., Jalkanen J.-P. (2014). Airborne emission measurements of SO_2_, NOx and particles from individual ships using a sniffer technique. Atmos. Meas. Tech..

[20] Alföldy B., Lööv J.B., Lagler F., Mellqvist J., Berg N., Beecken J., Weststrate H., Duyzer J., Bencs L., Horemans B. (2013). Measurements of air pollution emission factors for marine transportation in SECA. Atmos. Meas. Tech..

[21] Beecken J. (2015). Remote Measurements of Gas and Particulate Matter Emissions from Individual Ships. Ph.D. Thesis.

[22] Beecken J., Mellqvist J., Salo K., Ekholm J., Jalkanen J.-P., Johansson L., Litvinenko V., Volodin K., Frank-Kamenetsky D.A. (2015). Emission factors of SO_2_, NO_x_ and particles from ships in Neva Bay from ground-based and helicopter-borne measurements and AIS-based modeling. Atmos. Chem. Phys..

[23] Cheng Y., Wang S., Zhu J., Guo Y., Zhang R., Liu Y., Zhang Y., Yu Q., Ma W., Zhou B. (2019). Surveillance of SO_2_ and NO_2_ from ship emissions by MAX-DOAS measurements and the implications regarding fuel sulfur content compliance. Atmos. Chem. Phys..

[24] Kattner L., Mathieu-öffing B., Burrows J.P., Richter A., Schmolke S., Seyler A., Wittrock F. (2015). Monitoring compliance with sulfur content regulations of shipping fuel by in situ measurements of ship emissions. Atmos. Chem. Phys..

[25] Pirjola L., Pajunoja A., Walden J., Jalkanen J.-P., Rnkkö T., Kousa A., Koskentalo T. (2014). Mobile measurements of ship emissions in two harbour areas in Finland. Atmos. Meas. Tech..

[26] Zhang Y., Deng F., Man H., Fu M., Lv Z., Xiao Q., Jin X., Liu S., He K., Liu H. (2019). Compliance and port air quality features with respect to ship fuel switching regulation: A field observation campaign, SEISO-Bohai. Atmos. Chem. Phys..

[27] Alföldy B., Lööv J.B., Lagler F. (2011). Final Report on: Remote Sensing of Ships’ Emissions of Sulphur Dioxide, European Commission, Joint Research Centre. https://environment.ec.europa.eu/topics/air_en.

[28] Mellqvist J., Conde V., Beecken J., Ekholm J. (2017). Certification of an Aircraft and Airborne Surveillance of Fuel Sulfur Content in Ships at the SECA Border, CompMon. https://compmon.eu/.

[29] Villa T.F., Gonzalez F., Miljievic B., Ristovski Z.D., Morawska L. (2016). An Overview of Small Unmanned Aerial Vehicles for Air Quality Measurements: Present Applications and Future Prospective. Sensors.

[30] Ning Z. Application of UAV based sensing technology for ship emission and high sulfur fuel screening. Proceedings of the 4th International Symposium on Environment and Health.

[31] Ning Z. Integrated UAV and microsensing technology for ship emission and high sulphur fuel screening. Proceedings of the Motor Vehicle/Vessel Emissions Control Workshop 2019.

[32] Contini D., Gambaro A., Donateo A., Cescon P., Cesari D., Merico E., Belosi F., Citron M. (2015). Inter-annual trend of the primary contribution of ship emissions to PM_2.5_ concentrations in Venice (Italy): Efficiency of emissions mitigation strategies. Atmos. Environ..

[33] Gao Y., Lee S.C., Huang Y., Chow J.C., Watson J.G. (2016). Chemical characterization and source apportionment of size resolved particles in Hong Kong sub-urban area. Atmos. Res..

[34] (2019). THE SCIPPER PROJECT: D2.1 Review of Available Remote Systems for Ship Emission Measurements. https://www.scipper-project.eu/wp-content/uploads/2020/01/scipper_d2_1_20191220.pdf.

[35] Abhishek A., Peng W., Nirmal K.G., Li S. (2020). Protocol development for real-time ship fuel sulfur content determination using drone-based plume sniffing microsensor system. Sci. Total Environ..

